# Mobile Telehealth Intervention to Support Care Partners of Patients With Alzheimer Disease and Related Dementias (I-CARE 2): Protocol for a Randomized Effectiveness Clinical Trial

**DOI:** 10.2196/73387

**Published:** 2025-09-03

**Authors:** Jordan R Hill, Bailey Gardner, Miriam J Rodriguez, Lilian Golzarri-Arroyo, Roger S Zoh, Zayn Boustani, Jacqueline Burnell, Evan J Jordan, Malaz A Boustani, Richard J Holden

**Affiliations:** 1 Department of Health & Wellness Design School of Public Health - Bloomington Indiana University Bloomington United States; 2 Department of Epidemiology and Biostatistics School of Public Health - Bloomington Indiana University Bloomington United States; 3 Department of Medicine Indiana University School of Medicine Indianapolis United States; 4 Indiana University Center for Aging Research Regenstrief Institute Indianapolis, IN United States; 5 Center for Health Innovation and Implementation Science Indiana University School of Medicine Indianapolis, IN United States

**Keywords:** Alzheimer disease, dementia, behavioral and psychological symptoms of dementia, caregivers, technology, mobile telehealth intervention, protocol, randomized controlled trial

## Abstract

**Background:**

Nearly all individuals with Alzheimer disease and related dementias (ADRD) experience behavioral and psychological symptoms of dementia (BPSD), which include symptoms such as agitation, wandering, delusions, and hallucinations. Care partners (a person, often a family member, who provides care and support to someone with ADRD) struggle to manage BPSD, making symptom management a critical focus of intervention research. Our team has developed a mobile telehealth intervention (Brain CareNotes) to help care partners manage BPSD.

**Objective:**

This paper outlines a protocol for a randomized controlled trial of 160 care partners of patients with ADRD to test the effect of Brain CareNotes on care partner burden and patients’ BPSD.

**Methods:**

Participants will be recruited from partner health systems in Indiana, United States, by word of mouth, and through community outreach efforts. They will be randomly assigned to use either the Brain CareNotes mobile app intervention—which helps care partners manage BPSD through the support of a care coach, written and visual materials on developing care skills, and BPSD measurement and tracking—or an education-only mobile app control for 12 months. Data will be collected over the phone at baseline, 6 months, and 12 months. Primary outcomes assessed for this trial are (1) care partner burden and (2) patients’ BPSD. Secondary and exploratory outcomes assessed include care partner depressive symptoms, patient and care partner acute care use, intervention usability and acceptability, and intervention use. The trial will also collect patient and care partner demographics and measure care partner self-efficacy, care partner social support, patients’ ADRD severity, and patients’ functional abilities.

**Results:**

As of March 2025, we have enrolled 159 participants, and 42 have successfully completed the study.

**Conclusions:**

Brain CareNotes is a unique mobile app intervention to support care partners in managing BPSD. Due to the scalability of mobile health interventions, if Brain CareNotes is shown to be effective in reducing caregiver burden and patients’ BPSD, it has the potential for widespread adoption to support many ADRD care partners.

**International Registered Report Identifier (IRRID):**

DERR1-10.2196/73387

## Introduction

The National Plan to address Alzheimer disease and related dementias (ADRD) identifies behavioral and psychological symptoms of dementia (BPSD) as a critical focus of intervention research [1]. BPSD include both behavioral (eg, agitation, aggression, and wandering) and psychological (eg, delusions and hallucinations) symptoms [1]. Almost everyone with ADRD will experience BPSD, yet care partners (nonprofessional caregivers who are often family members of the person with ADRD) report having unmet needs for support with BPSD [2-5]. BPSD are often poorly managed, increasing the risk of avoidable hospitalizations, morbidity, mortality, and nursing home placement [4,6-12]. Care partners struggle to manage BPSD on their own, especially as conditions and symptoms change, leading to increased care partner stress, depression, and risk of mortality [12-15].

The PREVENT randomized controlled trial (R01HS010884) developed and evaluated a BPSD intervention that included care partner–facing materials and BPSD measures [16]. The intervention significantly reduced care partner burden and patients’ BPSD after 12 months when compared with augmented usual care [16]. The PREVENT intervention was then translated into a self-sustained clinical service called the Aging Brain Care clinic [17-20], which demonstrated the ability to hire, train, and use empathetic, nonclinically trained community health workers as care coaches to assist care partners with BPSD management. The Aging Brain Care clinic has had positive effects on BPSD and caregiver burden outcomes [18], but there are barriers to implementing a clinic-based intervention at scale (eg, reliance on in-person encounters, limited clinic operating hours, and the need to employ local clinicians) [17-22].

The National Institute on Aging’s published priorities call for technological interventions for ADRD care [23]. Remote technology (hereafter referred to as telehealth) solutions are especially desired because they can substitute for or extend clinic-based programs, which are costly, hard to scale, and hampered by rising staffing shortages [24]. With record rates of telehealth and mobile app use, now is the time to rigorously investigate technology-based remote interventions for managing BPSD.

Our team developed Brain CareNotes, which distills the Aging Brain Care Model [16,17] into a secure mobile telehealth app for care partners to manage BPSD with the aid of a remote care coach. It was professionally developed in collaboration with care partners, clinical staff, and leaders in the Aging Brain Care clinic but designed for scalability and broad use across settings. To our knowledge, Brain CareNotes is the first app of its kind [25] and delivers remote care support, BPSD measurement and tracking, psychoeducation, and person-centered care planning. Brain CareNotes was pilot tested with 53 care partners of patients with ADRD [26,27] and was demonstrated to be usable and acceptable by care partners. Although not statistically significant, the pilot test also indicated that Brain CareNotes may be effective in reducing care partner burden and patients’ BPSD.

**Figure 1:**
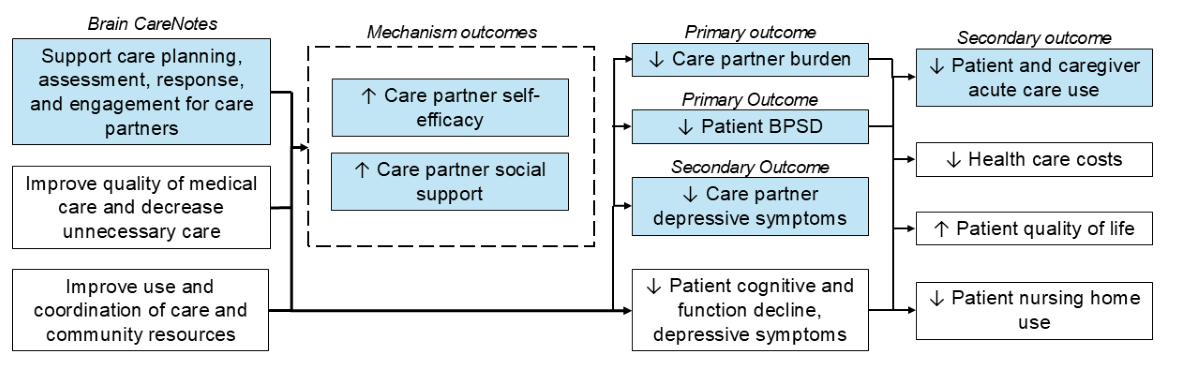
I-CARE 2 conceptual model. BPSD: behavioral and psychological symptoms of dementia.

The aim of this study is to test the effectiveness of Brain CareNotes in a larger-scale, randomized, parallel group clinical trial. Figure 1 depicts the study’s conceptual model (shaded sections are investigated in the current trial) based on the best-practice models of ADRD care [28,29]. Like PREVENT and the Aging Brain Care clinic, we anticipate that Brain CareNotes will decrease care partner burden and patients’ BPSD (study primary outcomes), while also lowering care partner depressive symptoms (study secondary outcome). It is hypothesized that improvements in these outcomes will also decrease the acute care use of both patients with ADRD and their care partners (secondary outcome). Care partner self-efficacy and social support are also being measured as the hypothesized primary social-behavioral mechanisms mediating the efficacy of Brain CareNotes [30,31].

## Methods

### Study Design

This study is a randomized controlled trial of 160 ADRD care partners to evaluate the real-world efficacy of Brain CareNotes on the primary outcomes of care partner burden and patients’ BPSD; patients with ADRD are not enrolled in the study, only their care partners are enrolled. Participants will be recruited from 2 health systems, by word of mouth, and from community outreach efforts and randomized to receive either the Brain CareNotes intervention or an education-only attention control app. Participants will remain in the study for 12 months.

The primary objectives of this trial are to test the effect of Brain CareNotes on (1) care partner burden and (2) patients’ BPSD after 12 months of use. Both primary outcomes will be assessed using the care partner-reported Neuropsychiatric Inventory (NPI) [32,33].

The secondary objectives are to test the effect of Brain CareNotes on (1) care partner depressive symptoms and (2) patient and care partner acute care use at 12 months. For care partner depressive symptoms, we will use the researcher-administered 9-item Patient Health Questionnaire-9 [34,35]. Acute care use data will be obtained from participant self-reports to identify both their and the patients’ hospital and emergency room visits that occurred within 12 months of enrollment.

To measure the predicted social-behavioral mechanisms mediating the efficacy of Brain CareNotes, we will also measure (1) care partner self-efficacy and (2) care partner social support at baseline, 6 months, and 12 months. These are measured using the Revised Scale for Caregiving Self-Efficacy [36] and the Multidimensional Scale of Perceived Social Support (MSPSS) [37], respectively.

App use, acceptability, and usability will also be obtained from participant self-reports at 6 and 12 months. App usability will be assessed using the Simplified System Usability Scale [38], and app acceptability will be assessed using the Behavioral Intention Questionnaire [39-41]. To track participant and patient characteristics, we will collect demographics for both the participant and the patient and assess patient functional abilities and dementia stage by having the participant complete the Functional Activities Questionnaire (FAQ) [42] and the Quick Dementia Rating System (QDRS) [43]. We will also share an app use questionnaire consisting of 2 questions: (1) “Over the last six months, how often have you used the app?” (2) “Over the last six months, when you have accessed the app, how long have been on it?”

Study outcomes are summarized in Table 1.

**Table 1:** I-CARE 2 study outcomes.

Outcome type and domain			Measure
**Primary**			
	Care partner burden	NPI^a^ caregiver distress subscore	
	Patients’ BPSD^b^	NPI	
**Secondary**			
	Care partner depressive symptoms	PHQ-9^c^	
	Care partner and patient acute care use	Self-report hospital and emergency room visits	
**Mechanism**			
	Care partner self-efficacy	Revised Scale for Caregiving Self-Efficacy	
	Care partner social support	MSPSS^d^	
**Process**			
	App use	In-app activity logs (intervention only), app use questionnaire (both groups)	
	App acceptance	Behavioral Intention Questionnaire	
	App usability	Simplified SUS^e^	
**Patient characteristics**			
	Functional abilities	FAQ^f^	
	Dementia stage	QDRS^g^	
	Patient demographics	Participant report	
**Participant characteristics**			
	Care partner demographics	Self-report	

^a^NPI: Neuropsychiatric Inventory.

^b^BPSD: behavioral and psychological symptoms of dementia.

^c^PHQ-9: 9-item Patient Health Questionnaire.

^d^MSPSS: Multidimensional Scale of Perceived Social Support.

^e^SUS: System Usability Scale.

^f^FAQ: Functional Activities Questionnaire.

^g^QDRS: Quick Dementia Rating System.

### Setting and Participants

This study has a planned enrollment of 160 care partners of patients with ADRD. There will be 80 participants in the intervention arm and 80 in the attention control comparator. We will obtain lists of eligible care partners’ contact information from partner health systems in Indiana, United States, including (1) primary care clinics at Indiana University Health (a nonprofit academic health care system), (2) Healthy Aging Transition Services clinics at Community Health Network (a nonprofit health care system), and (3) Eskenazi Health (a public health care system with special emphasis on vulnerable populations in Indiana). Care partners will be identified either by searching patient databases for patients with an ADRD diagnosis (the care partner will be their contact person) or by working with health care professionals at specific clinics to identify potentially eligible care partners. We will also distribute study information at conferences, in communities of interest, and by word of mouth (Multimedia Appendix 1). In addition to these recruitment efforts, our study is listed in databases such as ClinicalTrials.gov and Indiana University’s All IN for Health study listings. These postings reach potential participants statewide, nationwide, and internationally. We will consider international participants on a case-by-case basis and take into account international research ethical standards.

### Sample Size

The sample size was determined to achieve at least 80% power to detect group differences on both primary outcomes: care partner burden and BPSD. Adequate power required 64 participants per group (or N=128 in total) based on previous work [16]. Conservatively estimating 75% retention, a target total of 160 was set.

### Intervention Description

**Figure 2:**
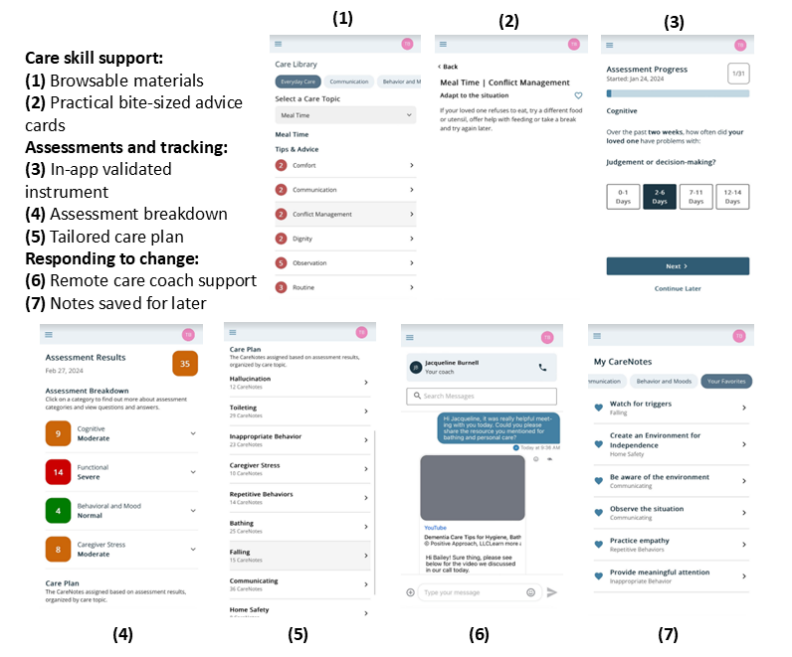
Brain CareNotes features.

The Brain CareNotes mobile telehealth app (Figure 2; [44]) is used by care partners for BPSD management. It includes remote communication with an external human support person—a care coach—as well as features for users to independently perform health-related activities (Textbox 1 [45,46]).

**Brain CareNotes mobile telehealth app features.**

- Care skill support features: users have 24/7 access to written and visual materials on developing care skills. These materials cover a range of topics, including (1) care partner burden, (2) understanding the patient with memory loss, (3) exercise, (4) personal care, (5) behaviors, (6) safety, and (7) delirium.
- Care coach support: each care partner user is assigned to a personal care coach employed by the study team. Care coaches operate remotely using a mobile phone or computer-based secure and HIPAA (Health Insurance Portability and Accountability Act)–compliant portal. Care coaches perform the following tasks daily: (1) monitor, respond to, and initiate messages to and from Brain CareNotes users; (2) address user activity, including new assessments; and (3) schedule and conduct biweekly or monthly phone calls with the care partner.
- Assessments and tracking: every 2 weeks, users are prompted to self-administer the Healthy Aging Brain Care Monitor [45,46]. Once an assessment is completed, results are immediately displayed in easy-to-read, color-coded visualizations with interpretation of whether scores are normal, mild, moderate, or severe. Past measurements can be displayed on demand to compare recent scores and assess change over time. Care coaches receive daily notifications when an assessment was completed or a message was received from a care partner.
- Responding to change over time: Brain CareNotes has bidirectional secure messaging, allowing unpaid care partner users to communicate back and forth with the care coach or for the care coach to initiate a conversation. Moreover, when a care partner reports a patient or care partner symptom, the app generates a set of specific CareNotes associated with each reported symptom.

Participants randomized to receive Brain CareNotes will be sent a HIPAA (Health Insurance Portability and Accountability Act)–compliant link to create an app account on their personal device. This researcher-generated link is required to gain access to Brain CareNotes. If the participant does not have a mobile device meeting the minimum technical requirements, a smartphone with an unlimited network data plan from AT&T, along with unlimited text messaging and pooled voice minutes, will be provided for the duration of the study.

An unmasked research assistant will guide the participant through loading the Brain CareNotes app or attention control app on the participant’s personal smartphone or on a smartphone provided by the study. The unmasked research assistant will provide written (and verbal, if requested) instructions on app use. They will provide as-needed technical assistance when requested in person or by phone and receive biweekly reminders to engage with their assigned app.

### Description of the Attention Control App

Participants randomized to the attention control group will receive the Dementia Guide Expert app, an existing BPSD mobile app freely available in the Google and Apple app stores. It was developed by the ENGAGE-IL initiative at the University of Illinois at Chicago (Health Resources and Services Administration grant U1QHP28730). Dementia Guide Expert provides education only, with no interactive BPSD management support, coaching, assessment, or external response. It contains “evidence-based expert information on what dementia is, types, contributing factors, risks, symptoms, stages, diagnosis, tests, treatment, management, communication techniques, and links to resources and support services” [47]. We will use an attention control comparator to mimic the intervention group’s use and access to technology, frequency and type of contact with research staff (ie, for installation and technical support), and expectancy of benefit [48].

### Inclusion and Exclusion Criteria

Eligibility will be assessed using a screening questionnaire (Textbox 2).

**Inclusion and exclusion criteria.**

**Inclusion criteria**

- Self-identified primary care partners of patients diagnosed with Alzheimer disease and related dementias (ADRD; at any stage) who are receiving primary care and community dwelling
- Participants are English literate

**Exclusion criteria**

- Participants cannot speak English
- They are involved in another trial that would prevent or interfere with study objectives (eg, another trial with an intervention aimed at reducing caregiver burden)
- Participants have a sensory or other impairment prohibiting the use of a mobile touchscreen device or another study activity (after correction).
- The patient with ADRD is a permanent resident of an extended care facility (eg, a nursing home, hospice facility, or locked memory unit of an assisted living facility).

### Recruitment and Retention

We will obtain lists of eligible care partners’ contact information from partner health systems or work with the health system to contact potentially eligible individuals as approved by the Indiana University Institutional Review Board (IRB), health system IRB (when applicable), and health system leadership. We will also obtain contact information for potential participants via word of mouth.

Research staff will call eligible care partners to determine if they are interested in study participation. Screening for eligibility will be conducted by phone or in person, when necessary, using a scripted questionnaire assessing eligibility. Data collection will occur remotely over the phone or videoconferencing software to minimize the burden on study participants. Research staff will also maintain engagement through regular contact with participants.

### Ethical Considerations

This trial is approved by the Indiana University IRB (15749) and is registered on ClinicalTrials.gov (NCT05733520). Indiana University Health system has approved the study through their internal approval system (they do not have an independent IRB), and the Community Health Network IRB has approved the distribution of study recruitment materials in their clinics. All protocol modifications will be approved by the IRB before implementation.

Informed consent will be obtained from participants by a research staff member. Individuals will receive verbal and written information about consent and offered a chance to have all their questions answered. Each participant will provide verbal documented consent over the phone.

As there will be no researcher interaction with the patient with ADRD or direct data collection from them, patients with ADRD will not be considered study participants or consented into the study.

Participants may withdraw from or discontinue the study at any time, including following the death of the patient with dementia or their placement in an extended care facility, by contacting a member of the research team. Participants may also be withdrawn, with notification, from the study to protect data integrity or if there is a danger to their or the research team’s safety related to continued participation. The risks of participation in this trial are expected to be minimal. Research personnel will be IRB approved and will have completed human subjects training, regular education, and updates on HIPAA regulations. Data will be stored in REDCap (Research Electronic Data Capture; Vanderbilt University), which is a widely used and secure browser-based, metadata-driven electronic data capture software solution and workflow methodology for designing clinical and translational research databases. Participants will be given a unique study ID to protect confidentiality and will not be identified in reports. Data collected through the Brain CareNotes app will be housed on HIPAA-compliant, secure cloud-based servers. Direct access to the app requires the user to input their email or phone number to which a one-time log-in code will be sent. After their first log-in, participants will also have the option to use their smartphone’s built-in biometric authentication (eg, fingerprint or facial recognition) to access the app. The only changes to log-in protocols will be those that result in equal or better security.

### Randomization

We will use a permuted block randomization scheme implemented within REDCap. Randomization will be stratified by recruitment source. Participants will be randomized after the baseline assessment by unmasked research staff. Research personnel masked to the randomization of individual participants will perform all primary and secondary data collection at the 6- and 12-month follow-ups. Participants will not be informed which app is the intervention and which is the control.

### Assessments and Outcomes

A research staff member will perform a baseline assessment with the participant before randomization and follow-up assessments at 6 and 12 months, as shown in Table 2. The research staff completing primary and secondary data collection for the 6- and 12-month follow-up assessments will be masked to the treatment condition. The research staff completing usability and acceptance data will be unmasked to the treatment condition. All assessments will be scheduled and conducted remotely over the phone or with videoconferencing software (eg, Zoom; Zoom Communications, Inc) or in person when necessary. Participants will receive US $25 per assessment period with an additional US $25 bonus if they complete all 3 assessment periods (for up to US $100 for the entire study).

**Table 2:** Assessment completion schedule.

	Baseline	Month 6	Month 12
Patient functional abilities (FAQ^a^)	✓		
Patient dementia stage (QDRS^b^)	✓		
Patient Care partner demographics	✓		
Neuropsychiatric Inventory	✓	✓	✓
Care partner depressive symptoms (PHQ-9^c^)	✓	✓	✓
Care partner acute care use		✓	✓
Patient acute care use		✓	✓
Care partner self-efficacy	✓	✓	✓
Care partner social support (MSPSS^d^)	✓	✓	✓
App usability (SUS^e^)		✓	✓
Behavioral Intention Questionnaire		✓	✓

^a^FAQ: Functional Activities Questionnaire.

^b^QDRS: Quick Dementia Rating System.

^c^PHQ-9: 9-item Patient Health Questionnaire.

^d^MSPSS: Multidimensional Scale of Perceived Social Support.

^e^SUS: System Usability Scale.

The primary outcomes assessed are (1) patients’ BPSD and (2) care partner burden. The secondary outcomes assessed are (1) care partner depressive symptoms and (2) patient and care partner acute care use.

Care partner burden and patients’ BPSD will be determined using the NPI [32,33], administered by research staff to the participant at baseline, 6 months, and 12 months. The NPI has been adopted by the Alzheimer’s Disease Cooperative Studies Group [49,50] to obtain information in 12 behavioral areas including delusions, apathy, hallucinations, disinhibition, agitation, depression, aberrant motor behavior, anxiety, nighttime behavior, and euphoria [32,33]. The NPI has both content and concurrent validity as well as between-rater, test-retest, and internal consistency reliability [32].

Care partner depressive symptoms will be assessed using the PHQ-9 [34,35], administered by research staff at baseline, 6 months, and 12 months. The PHQ-9 is a 9-item depression scale that has good internal consistency and test-retest reliability as well as convergent, construct, criterion, procedural, and factorial validity for the diagnosis of major depression [34,35,51-53].

We will also ask participants to self-report any acute care use in the preceding 6 months by themselves or the patient with ADRD during the 6- and 12-month follow-up visits.

### Additional Data Collection

To collect contextual information regarding the severity of the care recipient’s dementia, we will assess the care recipient’s functional abilities and dementia stage at baseline. The FAQ [42] and the QDRS [43] will be administered by research staff to the participant at baseline to assess the cognitive and functional status as well as the dementia severity of the patient with ADRD.

We will also collect patient and participant demographics at baseline, including age, sex, gender, race, ethnicity, education level, income, and living situation.

In addition to the primary and secondary outcomes, we will also measure (1) care partner self-efficacy, (2) care partner social support, (3) app acceptance, and (4) app usability throughout the study.

Care partner self-efficacy will be measured using the Revised Scale for Caregiving Self-Efficacy [36], and care partner social support will be measured by the MSPSS [37]. They will be administered by research staff to the participant at baseline, 6 months, and 12 months. Both scales have been validated in ADRD care partner samples, have strong psychometric qualities yet are brief, and measure constructs hypothesized and shown to act as the mechanisms of action for other psychosocial ADRD care partner interventions [30,31].

For both the control and intervention apps, we will assess app acceptance and app usability with surveys at the 6- and 12-month follow-ups. We will assess usability using the Simplified System Usability Scale [38], a 10-item questionnaire answered on a 5-point Likert scale (from strongly disagree to strongly agree). Technology acceptance will be assessed using the Behavioral Intention Questionnaire, a 4-item questionnaire using a 7-point response scale from 0 (not at all) to 6 (a great deal) [39,40].

### Analyses

At 12 months, the NPI Caregiver Distress scale will be compared between study groups using an analysis of covariance model with fixed effects for Brain CareNotes (vs attention control), health system, age, sex, race, recruitment source, and baseline values for NPI Caregiver Distress and NPI Total Score scales. For between-group comparisons, the difference in least squares means, corresponding SE, 2-sided 95% CIs, and 2-tailed *P* value will be derived from this analysis of covariance model and presented. Within-group change (least squares) means, corresponding SEs, 2-sided 95% CIs, and 2-tailed *P* values will also be presented. Standard model diagnostics will be performed to assess the validity of the proposed model; these diagnostics will include an examination of the residuals for normality, linearity, and homoscedasticity as well as testing for the significance of the intervention by baseline values interaction term to examine if intervention effect differs depending on the baseline levels of NPI Caregiver Distress or NPI Total Score scales.

The NPI Caregiver Distress scale will also be analyzed using a generalized linear mixed model (GLMM) [54,55] with an identity link function. GLMM allows us to analyze longitudinal data at each time point (baseline, 6 months, and 12 months) adjusting for multilevel dependencies within an individual (nested in the study group) and to explore additional covariates as mentioned earlier. Random intercepts, slopes, and fixed covariate effects will be estimated as needed. Time will be modeled as a continuous and categorical variable to model linear and nonlinear changes in care partner burden, respectively. The interaction between study group and time (ie, days between assessments) will be tested to determine if the rate of change in care partner burden is different between study groups. Standard model diagnostics will be performed to assess the validity of proposed models; these diagnostics will include examining the residuals for normality, linearity, and homoscedasticity and testing for the significance interaction term of the intervention by the baseline levels of the NPI Caregiver Distress and NPI Total Score scales. The variance-covariance structure of the random effects will be dependent on the variability among participants within groups. A preliminary test will be conducted to assess the significance of the homogeneity of variances [56] for within-group differences.

Analysis for patients’ BPSD and care partner depressive symptoms will be the same as for care partner burden, using the NPI Total Score scale and PHQ-9, respectively.

Acute care use will be analyzed as a dichotomous outcome (ie, yes or no for at least 1 emergency department or hospital visit—cumulative incidence) using a generalized linear model, specifically, a log-binomial regression model with fixed effects for Brain CareNotes (vs attention control), strata, and covariates described earlier. For the between–study group comparison, the estimated risk ratio (relative risk), SE, 95% Wald CI, and *P* value will be presented. Standard model diagnostics to assess final model validity and assumptions will be implemented.

As in the primary analysis, we will use a GLMM [54,55] with a log link function to model cumulative incidence. In addition, a Poisson model (GLMM with log link function and offset variable for follow-up duration) will be examined to model the incidence rate of acute care visit counts during the 12-month follow-up period.

The potential for 3-way and 2-way interactions will be examined to identify participant characteristics associated with increased benefit of the intervention. If statistically significant interactions are observed, additional intervention effect results will be presented as subgroup analyses to highlight intervention effect modifiers on primary and secondary outcomes separately.

Measures related to improved psychosocial outcomes (ie, social support and self-efficacy) will be tested as a potential mediator or moderators of intervention effects. We will also examine baseline characteristics (eg, sex and age) related to psychosocial outcomes as potential intervention effect moderators.

Similarly, mechanism measures (MSPSS and Revised Scale for Caregiving Self-Efficacy) will be evaluated for their potential mediation effect on the primary and secondary outcomes. We will also use investigate patient characteristics (FAQ or QDRS) as potential moderator. All analyses related to study primary, secondary, and mechanism outcomes will be performed by researchers masked to treatment condition.

Linear regression and classification and regression trees will be used to examine the effect of intervention timing, participant ratings on usability, acceptance, and actual use of technology on intervention efficacy.

### Handling Missing Data

Our general approach to missing data involves using all observed information while not exaggerating the precision of findings based on incomplete data [57,58]. All variables described for our final regression models will be used in our imputation procedures, that is, outcome measures, intervention condition, baseline outcome variables, strata variables (sex and race), age, and health system. The study group participant data will be imputed separately [59]. Multivariate imputation by chained equations methods using PROC MI and MIANALYZE procedures in SAS (SAS Institute) will be used to create multiple imputations for multivariate missing data (eg, continuous, binary, unordered categorical, and ordered categorical data) based on a fully conditional specification, where each incomplete variable is imputed by a separate model [60]. All missingness (nonresponse, attrition, or loss to follow-up) will be treated the same way for our benchmark analysis. Sensitivity analyses will be explored where reasons for attrition are accounted for in the generation of imputed covariate and outcome values. All variables (ie, outcome and baseline characteristics) described in the aforementioned models will be used for imputation procedures. The number of datasets to be imputed will be determined using the quadratic rule [61].

### Data and Safety Monitoring Plan and Board

The principal investigators (PIs), along with the independent data and safety monitoring board (DSMB), will be responsible for data and safety monitoring during the duration of the study. PIs will work to ensure the daily safety of participants, while the DSMB will advise PIs and National Institute on Aging program staff on the safety of participants, potential study risk and benefits, the ethicality of the study, scientific integrity, and participant recruitment.

The study team will meet with PIs on average at least every 2 weeks to discuss the ongoing progress of the study, data collection, and participant safety. Any reportable events will be discussed and reported within 48 hours (about 2 days). PIs will work will research staff to submit a written report to the DSMB every 6 months, unless otherwise recommended by the DSMB. This report will include study updates on the following: participant recruitment, accrual, randomization, and retention; treatment delivery and fidelity; data collection and quality; any adverse events that have occurred; and any protocol deviations that have occurred.

All participant data will be compiled and stored in the study’s HIPAA-compliant REDCap databases. Participant contact information will be collected from partner health system’s electronic databases and manually entered by the data management team. Participants’ survey responses, acute care use, and adverse events will be collected during assessment administration and entered directly into the database. App use will be estimated via app server queries that will be stored in IRB-approved databases. All data will be routinely reviewed for errors and cleaned by the data management team.

## Results

From May 2023 to March 2025, we have enrolled 159 participants, and 42 have successfully completed the study. Data analysis will begin once the final participant has completed their involvement in the study (estimated in spring 2026), and we anticipate results of these analyses will be published in late 2026 or early 2027. Results will be available to participants and the public on the study’s ClinicalTrials.gov (NCT05733520) page, and participants will be reminded of that link 1 month before their completion of the study.

## Discussion

### Anticipated Findings

This paper presents the protocol for a randomized controlled trial to test the efficacy of a mobile app to support care partners of patients with ADRD in managing BPSD. We hypothesize that, relative to the education-only attention control, care partners randomized to Brain CareNotes will have lower caregiver burden and will report lower care recipient BPSD. We also anticipate that care partners in the intervention group will report lower depressive symptoms, lower acute care use, increased self-efficacy, and increased social support.

Supporting care partners of patients with ADRD is a public health imperative [62]. BPSD occur commonly among patients with ADRD, and care partners often lack support for BPSD management [2-5], meaning there is a significant need for interventions to support care partners in this area. Mobile telehealth interventions—such as the intervention described in this study—are scalable solutions with the possibility of supporting large numbers of care partners in a variety of situations, for a lower cost. Brain CareNotes has the potential to be a scalable, effective intervention for BPSD management as it transforms other evidence-based interventions into a mobile app with the potential to be used by large numbers of care partners without constraints on geography, time of day, or clinic access.

### Limitations and Potential Challenges

There are some limitations and potential challenges to this study. First, while we have had success in recruiting participants on time, there are concerns about retaining participants in the study for the entire 12-month duration. Sample size was calculated assuming a 75% participant retention rate, as the 6-month pilot had 85% of participants complete the study. However, the likelihood of care recipient death or nursing home placement increases with the duration of the study, and this may impact our ability to retain as many participants as previously anticipated. In addition, our team does not have the ability to control the “dosing” of the intervention that participants receive, as they are able to use Brain CareNotes at their own discretion. This means that those randomized to the intervention group likely have differing app use patterns, which could affect its efficacy. While this is reflective of real-world use patterns of commercially available mobile apps, we are attempting to control this by sending app use reminders to participants in both the intervention and control groups every 2 weeks. Currently, Brain CareNotes is a website optimized for mobile viewing, so it is compatible with all mobile devices that can access the internet through a browser. In the future, it could be adapted into an app and made widely available on public app marketplaces (eg, Apple App Store and Google Play Store) for download onto a mobile device. While there are costs associated with Brain CareNotes once built (eg, technology maintenance and security and care coach compensation), it is likely that these costs are lower than other types of interventions. In addition, a mobile telehealth intervention like Brain CareNotes can remove some barriers to care partners (eg, geography) accessing BPSD support.

### Conclusions

Brain CareNotes distills the Aging Brain Care Model into a mobile app, and its historical BPSD tracking and care coach support features make it a unique mobile intervention to support care partners in managing BPSD. Due to the scalability of mobile health interventions, if Brain CareNotes is shown to be effective in reducing caregiver burden and patients’ BPSD, it has the potential for widespread adoption to support a large number of ADRD care partners.

## Appendix

Multimedia Appendix 1 Study information sheet.

Multimedia Appendix 2 Grant peer review comments from ZAG1 ZIJ-D (M2) - National Institute on Aging Special Emphasis Panel Dementia Care (National Institutes of Health, USA).

Multimedia Appendix 3 CONSORT-eHEALTH checklist (V 1.6.1).

Multimedia Appendix 4 SPIRIT checklist.

## Abbreviations

ADRD: Alzheimer disease and related dementias
BPSD: behavioral and psychological symptoms of dementia
DSMB: data and safety monitoring board
FAQ: Functional Activities Questionnaire
GAAIN: Global Alzheimer’s Associated Interactive Network
GLMM: generalized linear mixed model
HIPAA: Health Insurance Portability and Accountability Act
IRB: Institutional Review Board
MSPSS: Multidimensional Scale of Perceived Social Support
NPI: Neuropsychiatric Inventory
PHQ-9: 9-item Patient Health Questionnaire
PI: principal investigator
QDRS: Quick Dementia Rating System
REDCap: Research Electronic Data Capture

## Data Availability

Access to the study data will be facilitated in collaboration with the Global Alzheimer’s Associated Interactive Network (GAAIN). As a GAAIN Data Partner, data from this study will be shared in aggregate through the GAAIN portal [63].
