# Supplementary material for: Mobile Telehealth Intervention to Support Care Partners of Patients With Alzheimer Disease and Related Dementias (I-CARE 2): Protocol for a Randomized Effectiveness Clinical Trial
Source: JMIR Res Protoc. 2025 Sep 3;14:e73387. doi: 10.2196/73387 (PMC12444222; doi:10.2196/73387)
Supplement: Multimedia Appendix 1 [file resprot_v14i1e73387_app1.docx]

**INDIANA UNIVERSITY INFORMED CONSENT DOCUMENT FOR RESEARCH**

**I-CARE 2**

This informed consent applies to people serving as informal caregivers for individuals with Alzheimer’s disease or other related dementias (ADRD).

**Who is conducting the study?**

The study is being conducted by Drs. Richard Holden [[rjholden@iu.edu](mailto:rjholden@iu.edu)] and Malaz Boustani [[mboustan@iu.edu](mailto:mboustan@iu.edu)] of the Indiana University Schools of Public Health and Medicine. Support for the study is provided by the National Institute on Aging (NIA).

**What is the purpose of the study?**

The purpose of this study is to test the effects of a mobile health application on a) alleviating caregiver burden and b) improving the behavioral and psychological health of individuals with ADRD. About 160 patient-caregiver pairs will participate in this study.

**What will happen? How long will I** **be in the study?**

If you agree to participate today, you will do the following:

1. At the beginning of the study, after 6 months and after 12 months, you will be asked to schedule appointments with a research assistant to complete a series of assessments. At the six- and 12-month appointments, study personnel will ask you to report any hospital or emergency room visits you or the person you are caring for has made during the study period. These appointments will take no more than one hour and a half.
2. You will be randomized into one of two groups. Based on your assigned group, you will utilize a mobile health application or “app” that will either provide information to guide you through the essential elements of dementia care or pair you with a “care coach” who will help develop a care plan to address your caregiving challenges. You will be reminded to use the app at least monthly. If you do not own a smart phone, we will loan you a device free of charge. We will measure how you use the app and collect study-relevant data through the app during the duration of the study.
3. At the end of the study, we will contact you to schedule a final appointment. At that appointment we will collect the loaned devices or request that you delete the app from your personal phone.
4. Lastly, researchers may ask your permission to re-use the demographic information collected about you if you consent to participate in other studies the I-CARE 2 team is conducting.

**What are the risks of taking part in this study?**

This study uses third-party software. This study also includes the collection of information about you, so one risk to you is a possible loss of confidentiality. We cannot guarantee absolute confidentiality, but we will do everything possible to protect your information. This includes keeping your information in protected electronic databases that can only be accessed by research staff.

It is also possible that some of the questions asked within the apps and during assessments could cause discomfort or anxiety. While completing the assessments and surveys, you can tell the researcher that you feel uncomfortable or do not want to answer a particular question.

**What are the potential benefits of taking part in this study?**

Benefits to science and humankind might result from this study. ADRD and informal caregiver burden are each recognized as important public health problems. Strategies that improve our understanding of how to care for patients with ADRD and their informal caregivers are urgently needed.

You also might experience benefits from being in this study. Participation in this proposed study affords access to a greater level of health care with the potential for improved quality and outcomes of care.

**Will I be compensated for my participation?**

You will receive payment for taking part in this study. You will receive a $25 gift card each time you complete an appointment or a total of $100 in gift cards for completing all appointments. You will not receive payment for partially completed appointments.

**Is this study voluntary? What will happen if I decide not to be in this study?**

You may choose not to take part in the study. You may choose to leave the study at any time. Deciding not to participate, or deciding to leave the study later, will not result in any penalty or loss of benefits to which you are entitled. If you decide to withdraw, please contact the study staff at (XXX) XXX-XXXX or email [icare2@iu.edu.](mailto:icare2@iu.edu.)

Additionally, the PI may, with notification, withdraw participants from the study if there is a danger to their or the research team’s safety related to continued participation, or a threat to the integrity of the data collected.

**What happens if my caregiving situation changes?**

If your caregiving situation changes – for example, you are no longer a) the primary caregiver for the person you care for and/or b) a caregiver at all -- you may choose to continue to participate.

**Confidentiality and Privacy:**

For the protection of your privacy, this research is covered by a Certificate of Confidentiality from the NIA. The researchers may not disclose or use any information, documents, or specimens that could identify you in any civil, criminal, administrative, legislative, or other legal proceeding, unless you consent to it.  Information, documents, or specimens protected by this Certificate may be disclosed to someone who is not connected with the research:

1. if there is a federal, state, or local law that requires disclosure (such as to report child abuse or communicable diseases);
2. if you consent to the disclosure, including for your medical treatment;
3. if it is used for other scientific research in a way that is allowed by the federal regulations that protect research subjects;
4. for the purpose of auditing or program evaluation by the government or funding agency.

A description of this clinical trial will be available on [ClinicalTrials.gov](https://nam12.safelinks.protection.outlook.com/?url=http%3A%2F%2Fclinicaltrials.gov%2F&data=05%7C02%7Cgardnbai%40iu.edu%7C6643037e151b42be0c8408dd46dfcded%7C1113be34aed14d00ab4bcdd02510be91%7C1%7C0%7C638744649221756162%7CUnknown%7CTWFpbGZsb3d8eyJFbXB0eU1hcGkiOnRydWUsIlYiOiIwLjAuMDAwMCIsIlAiOiJXaW4zMiIsIkFOIjoiTWFpbCIsIldUIjoyfQ%3D%3D%7C0%7C%7C%7C&sdata=x1LKvrKdFTf5Z2ANTUtZnrhFkkNml1hTpy3f0lXOnuY%3D&reserved=0), as required by U.S. law. This website will not include information that can identify you. At most, the website will include a summary of the results. You can search this website at any time.

**Contact Information:**

- For concerns related to the study, contact Dr. Holden: (812) 856-6265, [rjholden@iu.edu](mailto:rjholden@iu.edu) or Dr. Boustani: (317) 274-8536, [mboustan@iu.edu](mailto:mboustan@iu.edu).
- For questions about the study, contact the research team: (812) 856-3047 or [icare2@iu.edu](mailto:icare2@iu.edu).
- For questions about your rights as a participant or to discuss problems with the study please contact the Indiana University Human Subjects Office at (800) 696-2949 or at [irb@iu.edu.](mailto:irb@iu.edu)

**Participant’s Consent:**

In consideration of all the above, I give my consent to participate in this research study. I will be given a copy of this informed consent document to keep for my records. I agree to take part in this study.
