# Supplementary material for: Mobile Telehealth Intervention to Support Care Partners of Patients With Alzheimer Disease and Related Dementias (I-CARE 2): Protocol for a Randomized Effectiveness Clinical Trial
Source: JMIR Res Protoc. 2025 Sep 3;14:e73387. doi: 10.2196/73387 (PMC12444222; doi:10.2196/73387)
Supplement: Multimedia Appendix 2 [file resprot_v14i1e73387_app2.pdf]

SUMMARY STATEMENT
(Privileged Communication)

PROGRAM CONTACT:
LISA ONKEN
(30I) 496-3131
lonken@nia.nih.gov

Release Date: **04/05/2022**
Revised Date:

Application Number: **1 R01 AG078234-01**

Principal Investigators (Listed Alphabetically):

BOUSTANI, MALAZ
HOLDEN, RICHARD J (Contact)

Applicant Organization: TRUSTEES OF INDIANA UNIVERSITY

Review Group: **ZAG1 ZIJ-D (M2)**
National Institute on Aging Special Emphasis Panel
Dementia Care

Meeting Date: **03/17/2022**
Council: **MAY 2022**
Requested Start: **07/01/2022**
RFA/PA: **PAR21-307**
PCC: **2BBCHLO**

Project Title: **I-CARE 2 RCT: Mobile Telehealth to Reduce Alzheimer's-related Symptoms for Caregivers and Patients**
SRG Action: **Impact Score:35**
Next Steps: **Visit https://grants.nih.gov/grants/next_steps.htm**
Human Subjects: 30-Human subjects involved - Certified, no SRG concerns
Animal Subjects: 10-No live vertebrate animals involved for competing appl.
Gender: 1A-Both genders, scientifically acceptable
Minority: 1A-Minorities and non-minorities, scientifically acceptable
Age: 3A-No children included, scientifically acceptable

Project Year	Direct Costs Requested	Estimated Total Cost
1	497,243	789,861
2	498,769	792,285
3	498,769	792,285
4	498,769	792,285
5	498,638	792,077
TOTAL	2,492,188	3,958,794

ADMINISTRATIVE BUDGET NOTE: The budget shown is the requested budget and has not been adjusted to reflect any recommendations made by reviewers. If an award is planned, the costs will be calculated by Institute grants management staff based on the recommendations outlined below in the COMMITTEE BUDGET RECOMMENDATIONS section.

RESUME AND SUMMARY OF DISCUSSION: This new application, for Dementia Care and Caregiver Support Intervention Research (R01, Clinical Trial Required) is tendered by Indiana University on behalf of Principal Investigator (PI) Dr. Richard Holden and Multiple Principal Investigator (MPI) Dr. Malaz Boustani. An MPI Leadership Plan is included. It requests five years of funding to establish an evidence-based intervention model for the management of behavioral and psychological symptoms of dementia, embed this evidence into the Brain CareNotes app, and set up a pilot study to examine its feasibility and potential efficacy. To accomplish this, the research team will conduct I-CARE 2, a Stage III randomized clinical trial as the next step in this work, which is based on the NIH Stage Model of Intervention Development, with the primary endpoint being a potential reduction of informal caregiver burden and behavioral and psychological symptoms of dementia at 12 months. If successful, this work has the potential to yield efficacy data regarding this scalable, non-pharmacological intervention for behavioral and psychological symptoms of dementia. This work builds on Dr. Holden's experience in technology intervention research applicable to Alzheimer's disease and related dementias (ADRD), and he is currently PI or site PI on four NIH-funded technology studies related to ADRD care or prevention. Together with Dr. Boustani, the MPI, he has overseen the original I-CARE clinical trial. Dr. Boustani is a geriatrician and senior health services researcher with experience using medical informatics and implementation science for the study of the care of people with ADRD. Thus, Drs. Holden and Boustani are ideally-suited to lead this research effort. Other notable strengths include the high significance of the research topic; strong and experienced team (with a collaboration with a strong healthcare system); inclusion of a diverse sample; good fidelity and monitoring, controls, outcomes; and analytic plan. However, several weaknesses temper enthusiasm, including lack of clarity regarding what mechanisms are being targeted for outcomes and how this work could be done when another group is leading the control arm, that the primary outcomes are not put into a broader context (.e.g., functional status of patients), lack of a rationale for the skill support feature, that there is no in-person initial contact to facilitate technology uptake, lack of power to detect sex and racial/ethnic differences, lack of inclusion of caregiver-patient relationship (e.g., living together or not) in the analysis, and a lack of control for multiple comparisons.

DESCRIPTION (provided by applicant): Among patients with Alzheimer's disease and related dementias (ADRD) and their informal caregivers, behavioral and psychological symptoms of dementia (BPSD) are a critical need requiring scalable, evidence- based intervention. As many as 97% of patients with ADRD will ultimately experience BPSD, yet they are poorly managed and remain the top source of caregiver burden. Technology may be a solution; indeed, the National Institute on Aging and others demand mobile technology-based behavioral interventions to support informal caregivers of patients with ADRD. Systematic reviews and market analyses of existing mobile technologies or "apps" demonstrate promise but critical limitations: lack of scientific foundation and evidence of efficacy; missing features and functions; and low to moderate quality. Our interdisciplinary team followed the NIH Stage Model for Behavioral Intervention Development to: 1) establish an evidence-based intervention model for BPSD management (NIH Model Stage 0); 2) apply user- centered design to embed this evidence-based model into Brain CareNotes, a mobile telehealth app (NIH Model Stage IA); and 3) conduct I-CARE, a set-up pilot study that established the feasibility and potential efficacy of Brain CareNotes (NIH Model Stage IB-II). The pilot study demonstrated that at the 6-month endpoint, Brain CareNotes reduced informal caregiver burden and reduced BPSD. Here we propose I-CARE 2, a Stage III randomized clinical trial (RCT), as the next step in the NIH Stage Model. I-CARE 2 will evaluate the real-world efficacy of Brain CareNotes on the primary outcomes of informal caregiver burden and BPSD at 12 months. We plan to enroll N=160 community-dwelling, English-speaking informal caregivers of patients with ADRD, across the state of Indiana. Informal caregivers will be randomized (stratified by sex and race) to 12 months of Brain CareNotes (n=80) or Attention Control education-only app (Dementia Guide Expert) (n=80). Follow-up will occur at 12 months, with additional assessments at 6 months to test for early effects. We will test primary hypotheses that, relative to Attention Control, informal caregivers randomized to Brain CareNotes will have: (H1) lower caregiver burden as measured by the Caregiver Distress sub-score on the Neuropsychiatric Inventory (NPI); and (H2) lower

BPSD as measured by the NPI Total Score. Secondary hypotheses will be tested comparing groups on (H3) depressive symptoms as measured by the Patient Health Questionnaire (PHQ)-9 and (H4) acute care utilization as determined by the number of hospital and emergency room visits captured in the statewide regional health information exchange. If successful, this NIH Stage III RCT study will yield evidence of the efficacy of a highly scalable non- pharmacological intervention for BPSD, one of the most burdensome aspects of ADRD care. If our caregiver- facing mobile telehealth app is efficacious in real-world settings, subsequent Stage IV-V effectiveness and implementation research efforts can help relieve the critical public health burden of ADRD.

PUBLIC HEALTH RELEVANCE: This randomized clinical trial tests how telehealth technology can positively affect informal caregivers and patients living with Alzheimer's disease and related dementias. The project will gather data over time to measure the impact of our evidence-based Brain CareNotes mobile telehealth app on informal caregiver burden and patients' behavioral and psychological symptoms of dementia, in a diverse Indiana-wide sample.

DISCLAIMER: Please note that the following critiques were prepared by the reviewers prior to the Study Section meeting and are provided in an essentially unedited form. While there is opportunity for the reviewers to update or revise their written evaluation, based upon the group's discussion, there is no guarantee that individual critiques have been updated subsequent to the discussion at the meeting. Therefore, the critiques may not fully reflect the final opinions of the individual reviewers at the close of group discussion or the final majority opinion of the group. Thus, the Resume and Summary of Discussion is the final word on what the reviewers actually considered critical at the meeting.

CRITIQUE 1:

Significance: 1
Investigator(s): 1
Innovation: 2
Approach: 2
Environment: 1

Overall Impact:
The significance of this application is high. The team is well-qualified to conduct the proposed project. The project builds directly on the team's systematic line of research, guided by the NIH Stage Model of Intervention Development. Innovation is modest. The approach is generally very strong, with a few minor concerns. Overall, this is an excellent application.

1. Significance:
Strengths
- The significance of this application is high.
- Alzheimer's disease (AD) is a highly prevalent and debilitating medical condition.
- 97% of persons with AD experience behavioral and psychological symptoms of dementia (BPSD), presenting a significant burden on informal caregivers and jeopardizing their health and quality of life (including stress, depression, and risk for mortality).
- The proposed project appears highly responsive to the NIA's request for projects that leverage digital technology to support unmet needs of caregivers.

Weaknesses
- None identified.

2. Investigator(s):
Strengths

- The team has extensive experience designing and evaluating technology for older adults and caregivers.
- The team has considerable expertise in AD and dementia care, and working with caregivers.
- The team has extensive experience with developing and testing the intervention to be evaluated in the proposed trial, via both traditional person-delivered models of intervention and, more recently, via a mobile version of the model.
- Overall, the team appears well-suited to conduct the proposed project.

Weaknesses
- None identified.

3. Innovation:
Strengths
- The applications states that the proposed project is innovative because it evaluates the first caregiver-facing mobile app based on a proven model of care for BPSD management (The Aging Brain Care Model).
- The application also indicates the proposed project is innovative because the mobile app has a broader array of features/functions to support symptom management relative to other apps (such as symptom tracking functionality and the ability to have bi-directional messaging between a caregiver and a clinician or care coach).
- The proposed project has prioritized inclusion of a diverse sample (as they did in their pilot work), which adds to the innovation of the proposed project relative to other similar projects.

Weaknesses
- There are several other mobile apps for caregivers targeting BPSD in various stages of testing and development, which somewhat reduces innovation.
- The proposed trial largely incrementally builds on the team's prior Stage IB/II trial (and has a similar design, metrics, and sites).

4. Approach:
Strengths
- A technology-based support system for caregivers increases the reach and scalability of support.
- This line of research is systematic and follows the NIH Stage Model of Intervention Development.
- The partnership with Eskenazi Health, the nation's second-largest safety net health system, is a strength, and may facilitate racial, economic, and geographic diversity in the sample.
- The data from a prior trial evaluating a non-app-based version of the Aging Brain Care model showed promise.
- Solid fidelity and monitoring procedures.
- The attention control group is justified.
- Outcome measures are well-considered.
- Planned analyses are strong.

Weaknesses
- Details about why each of the 4 components of the intervention (care skills; assess and monitor symptoms over time; respond in a timely and individualized manner; and engage caregivers) are considered the core ingredients of the therapeutic approach, are missing. How do they impact targeted mechanisms and outcomes, and what is the empirical support for each of the four key ingredients?
- Research staff will reportedly help participants register an account, provide software/ hardware training and user manuals, and troubleshoot technical issues. Can this be done at the same level for the attention group app that the team does not control versus the app they developed and can control?

- The application does not describe why the investigators think decreased caregiver/patient symptoms are the putative mechanism by which downstream endpoints improve. What empirical support do they have for this? This issue is important given that caregiver and patient symptoms are the key outcomes in the proposed trial.

5. Environment:
Strengths
- Strong.

Weaknesses
- None identified.

Study Timeline:
Strengths
- Appears reasonable. The team's success in completing their Stage IB/II study on time underscores confidence in their ability to complete the proposed trial in accordance with the proposed timeline.

Weaknesses
- None identified.

Protections for Human Subjects:
Acceptable Risks and/or Adequate Protections.
- Acceptable.

Data and Safety Monitoring Plan (Applicable for Clinical Trials Only):
Acceptable.
- They also propose a Data Safety and Monitoring Board.

Inclusion Plans:
- Sex/Gender: Distribution justified scientifically.
- Race/Ethnicity: Distribution justified scientifically.
- For NIH-Defined Phase III trials, Plans for valid design and analysis: Scientifically acceptable.
- Inclusion/Exclusion Based on Age: Distribution justified scientifically.
- Acceptable.

Resource Sharing Plans
Acceptable.

Budget and Period of Support
Recommend as Requested

CRITIQUE 2:

Significance: 2
Investigator(s): 1
Innovation: 2
Approach: 4
Environment: 2

Overall Impact:
This is a Stage III randomized clinical trial designed to evaluate the real-world efficacy of Brain CareNotes, a smart phone-based intervention, on the primary outcomes of informal caregiver burden

and BPSD at 12 months. A talented, productive, and experienced research team with extensive research infrastructure at Indiana University will carry out this study. The intervention is based on successful pilot studies completed by the applicants. The proposed intervention is innovative, with the potential of being easily scaled up if it is successful. The overall approach taken to implement this study is strong, but it also raises a number of issues and concerns that could have been addressed more effectively. The conceptual model for this study, along with the discussion of mechanisms of action, is confusing. Specifically, the mechanisms through which the intervention, or its four components, affects the primary outcome, BPSD, is not explained. Several important covariates, such as the functional status of the patient, are not measured and, therefore, not analyzed. The stratification strategy leads one to expect that the intervention might be evaluated within sex/race groups, but this is not the case. The study is not powered to address diversity issues in a meaningful way. Overall, the flaws listed here and below are not fatal, but they significantly detract from an otherwise good application.

1. Significance:
Strengths
- The application is responsive to multiple NIA dementia care initiatives.
- If successful, the intervention strategy is potentially scalable.
- May improve well-being of caregivers and care recipients and impact health care utilization of persons with AD.

Weaknesses
- Not responsive to mechanisms criterion.

2. Investigator(s):
Strengths
- Drs. Richard Holden and Malaz Boustani will serve as Principal Investigator and Multiple Principal Investigator, respectively. Dr. Holden has a strong track record for developing and applying systems engineering theories, tools, and methods to design, implement, and evaluate human-centered technology interventions. He has a strong track record of relevant publications and externally funded grants.
- Dr. Boustani is a geriatrician and senior health services investigator, with extensive experience using medical informatics and implementation science to transform brain care, including care of AD patients. He has a strong record of publications and externally funded grants relevant to the proposed study.
- The research team includes strong expertise in project management, biostatistics, systems engineering, AD care, and participant recruitment.

Weaknesses
- None noted.

3. Innovation:
Strengths
- Brain CareNotes (intervention) is an innovative mobile strategy that focuses specifically on BPSD symptoms of dementia. It addresses clinician shortages by using technology. The investigators have marketed a version of the app on the Apple/Android app store.
- The intervention is relatively long in duration (12 months) as compared to other intervention studies.
- Diverse gender/race samples will be recruited, although minority samples are too small to permit sub-group analyses.

Weaknesses
- Uptake and utilization of mobile caregiving apps has been problematic (low), as reported in the literature. There is a need for innovative strategies to assure uptake, which are not present in this application.

4. Approach:
Strengths
- Strong evidence base for the proposed intervention.
- The addition of patient acute care utilization over 12 months is a plus.
- Good fidelity monitoring program.
- Care coach is important component of the intervention.
- Appropriate analysis plan, but stratification variables are treated as an afterthought (see below).

Weaknesses
- The application states that disturbances in behavior and affect, including agitation, depression, aggression, and apathy or sleep disturbance, are the most prevalent sources of burden for caregivers. There is no discussion of how these symptoms fit within a broader array of risk factors for adverse outcomes for caregivers/care recipients (e.g., sociodemographic factors, access to care, functional status and pragmatics of care provision, hours of care provided, etc.). For example, the functional status of the patient (types and amount of care they need) is a major driver of caregiver outcomes. Will this be measured?
- Will living with the care recipient versus not be used as an eligibility criterion? If not, will it be measured and used in the analysis? The application mentions ”living situation” will be measured, but it is not clear what this means.
- The skill support feature of the intervention is a hodgepodge of strategies that have been used in other trials. The rationale for the specific features listed is not clear.
- The conceptual model is confusing. It shows “how Brain CareNotes implements the four “key ingredients” of BPSD management and thus affects our primary outcomes, secondary outcomes, and other downstream outcomes. Decreased caregiver/patient symptoms are the putative mechanism by which downstream endpoints improve.” Since BPSD is the primary outcome of the study, the model needs to explain how the intervention, or its components, affect(s) BPSD. The conceptual basis for this is not presented. Moreover, no attempt is made to tease out, conceptually or analytically, the relative impact of the components of the intervention on BPSD and caregiver burden/depression. The model presented also ignores important interrelationships between patient and caregiver outcomes.
- What is the functional value/impact of each component of the intervention, and will any effort be made to assess the impact of component elements?
- Lack of initial, in-person contact to introduce the intervention and provide training on use of the mobile app is likely to lead to low uptake. Will remedial follow-up procedures be used for individuals who do not use the phone/intervention?
- The study is not powered to detect within-group sex/race differences. For example, 14 Black/African American males will be allocated, leaving 7 persons per group. Ethnicity/race will be used as covariates along with other variables.
- The relationship between caregiver and care recipient and living with versus not, should be added to the covariate list, as they are important drivers of outcomes.
- If randomization is successful, there should be no baseline differences in outcome variables by groups. Why adjust for baseline outcome variables in the Analysis of Covariance?

5. Environment:
Strengths
- A broad array of clinical and research infrastructure resources are identified, including the Indiana Clinical and Translational Sciences Institute, academic departments in Indiana University’s Schools of Public Health and Medicine, the Eskenazi Health system, the community-based partner Indianapolis Discovery Network for Dementia, and the Indiana AD Research Center.
- These resources provide a strong base for participant recruitment, project execution and analysis, and dissemination.
- Letters of support from key partners are provided.

Weaknesses

- Although the support infrastructure is impressive, there is relatively little specificity as to how each of the listed resources will contribute to the proposed study.

Study Timeline:
Strengths

- Study timeline is appropriate.

Weaknesses

- None noted.

Protections for Human Subjects:
Acceptable Risks and/or Adequate Protections.

- Risks and benefits are adequately described.

Data and Safety Monitoring Plan (Applicable for Clinical Trials Only):
Acceptable.

- A detailed plan is presented, including specification of adverse events.

Inclusion Plans:

- Sex/Gender: Distribution justified scientifically.
- Race/Ethnicity: Distribution justified scientifically.
- For NIH-Defined Phase III trials, Plans for valid design and analysis: Scientifically acceptable.
- Inclusion/Exclusion Based on Age: Distribution not justified scientifically.
- "Adults" will be recruited. It is not clear why younger ages are excluded.

Budget and Period of Support
Recommend as Requested

CRITIQUE 3:

Significance: 2
Investigator(s): 2
Innovation: 5
Approach: 5
Environment: 1

Overall Impact:
Drs. Holden and colleagues propose to conduct a clinical trial employing their app-based health intervention program (Brain CareNotes) to evaluate its real-world efficacy on the primary outcomes of informal caregiver burden and BPSD at 12 months. Aim 1 will examine the effects on Brain CareNotes on caregiver burden and Aim 2 will examine the effects of patient BPSD at 12 months. Secondary aims will test the effect of Brain CareNotes on caregiver and patient acute care utilization and depressive symptoms at 12 months. The group has an established track record for performing this type of work, although it is unclear how mechanisms will be identified of the study. The study is not very innovative, although the scalability of the app-based approach is attractive.

1. Significance:
Strengths

- Dementia and dementia caregiver well-being is of critical importance.

Weaknesses

- This study will address the effects of the Brain CareNotes app, but the supporting mechanisms have yet to be identified.

2. Investigator(s):
Strengths
- Strong group of investigators with a track record of carrying out this work.

Weaknesses
- Not provided.

3. Innovation:
Strengths
- The use of a tailored, app-based delivery method derived from stage 0 preliminary findings are attractive.

Weaknesses
- The use of the app with a control group does not exhibit novelty or innovation.

4. Approach:
Strengths
- Well-designed and written application.

Weaknesses
- It is not clear if multiple comparisons are considered.
- Mediation modeling to identify psychological mechanisms of action is missing but needed.
- No mechanisms are proposed.

5. Environment:
Strengths
- Excellent environment.

Weaknesses
- None noted.

Study Timeline:
Strengths
- Well-organized.

Weaknesses
- None noted.

Protections for Human Subjects:
Acceptable Risks and/or Adequate Protections.

Data and Safety Monitoring Plan (Applicable for Clinical Trials Only):
Acceptable.

Inclusion Plans:
- Sex/Gender: Distribution justified scientifically.
- Race/Ethnicity: Distribution justified scientifically.
- Inclusion/Exclusion Based on Age: Distribution justified scientifically.

Resource Sharing Plans:
Acceptable.

Budget and Period of Support:
Recommend as Requested.

THE FOLLOWING SECTIONS WERE PREPARED BY THE SCIENTIFIC REVIEW OFFICER TO SUMMARIZE THE OUTCOME OF DISCUSSIONS OF THE REVIEW COMMITTEE, OR REVIEWERS' WRITTEN CRITIQUES, ON THE FOLLOWING ISSUES:

PROTECTION OF HUMAN SUBJECTS: ACCEPTABLE. Acceptable Risks and/or Adequate Protections are described in the application.

INCLUSION OF WOMEN PLAN: ACCEPTABLE. Of the 180 human subjects, 79% are women.

INCLUSION OF MINORITIES PLAN: ACCEPTABLE. Of the 180 human subjects, 31% are underrepresented minorities.

INCLUSION ACROSS THE LIFESPAN: ACCEPTABLE. The target population for this study is adults and older adults.

COMMITTEE BUDGET RECOMMENDATIONS: The budget was recommended as requested.

Footnotes for 1 R01 AG078234-01; PI Name: Holden, Richard J

NIH has modified its policy regarding the receipt of resubmissions (amended applications).See Guide Notice NOT-OD-18-197 at https://grants.nih.gov/grants/guide/notice-files/NOT-OD-18-197.html. The impact/priority score is calculated after discussion of an application by averaging the overall scores (1-9) given by all voting reviewers on the committee and multiplying by 10. The criterion scores are submitted prior to the meeting by the individual reviewers assigned to an application, and are not discussed specifically at the review meeting or calculated into the overall impact score. Some applications also receive a percentile ranking. For details on the review process, see http://grants.nih.gov/grants/peer_review_process.htm#scoring.

MEETING ROSTER

The roster for this review meeting is displayed as an aggregated roster that includes reviewers from multiple AG Special Emphasis Panels of the NIA SEP Aggregate roster for May 2022 Council for the 2022/05 council round.
This roster for AG is available here.
